# Epigenome-wide association study detects a novel loci associated with central obesity in healthy subjects

**DOI:** 10.1186/s12920-021-01077-9

**Published:** 2021-09-23

**Authors:** Ting Xie, Vesna Gorenjak, Maria G. Stathopoulou, Sébastien Dadé, Eirini Marouli, Christine Masson, Helena Murray, John Lamont, Peter Fitzgerald, Panos Deloukas, Sophie Visvikis-Siest

**Affiliations:** 1 INSERM UMR U1122, IGE-PCV, Faculté de Pharmacie, Université de Lorraine, 30 Rue Lionnois, 54000 Nancy, France; 2 Queen Mary University of London, London, UK; 3 Randox Laboratories Ltd, Crumlin, UK; 4 Present Address: ‘Université Côte d’Azur’, INSERM U1065, C3M, 06204 Nice, France; 5 Present Address: CRCT, INSERM U1037, 31037 Toulouse, France; 6 Present Address: Université Paul Sabatier III’, 31400 Toulouse, France

**Keywords:** Central obesity, Methylation, EWAS, Epigenetics

## Abstract

**Background and aims:**

Central obesity is a condition that poses a significant risk to global health and requires the employment of novel scientific methods for exploration. The objective of this study is to use DNA methylation analysis to detect DNA methylation loci linked to obesity phenotypes, *i.e*. waist circumference and waist-to-hip ratio adjusted for BMI.

**Methods and results:**

Two-hundred and ten healthy European participants from the STANISLAS Family Study (SFS), comprising 73 nuclear families, were comprehensively assessed for methylation status using Illumina Infinium HumanMethylation450 BeadChip. An epigenome-wide association study was performed, which identified a CpG site cg16170243 located on chromosome 18q21.2 significantly associated with waist circumference, after adjusting for BMI (β = 2.32, SE = 0.41, P_adj_ = 0.048). Cg16170243 corresponds to a 50 bp-length human methylation oligoprobe located within the *AC090241.2* gene that overlaps *ST8SIA5* gene. No significant association was observed with waist-to-hip ratio adjusted for BMI (P_adj_ > 0.05).

**Conclusions:**

A novel association between DNA methylation and WC was identified, which is demonstrating that epigenetic mechanisms may have a significant impact on waist circumference ratio in healthy individuals. Further studies are warranted to address the causal effects of this association.

**Supplementary Information:**

The online version contains supplementary material available at 10.1186/s12920-021-01077-9.

## Background

Epigenetic changes are covalent modifications of cytosine bases, histones and changes in nucleosome positioning that can modulate the effect of a genotype on a particular phenotype and thus affect physiological mechanisms as well as the pathophysiology of many diseases [1]. The most widely studied among them is cytosine methylation [2], a covalent attachment of methyl group to a DNA sequence that generally results in silencing of genes encoded in the methylated region [3]. DNA methylation patterns are not static but undergo precise, highly coordinated changes that can be mediated both by environmental and genetic factors and inherited through mitotic cell divisions [4]. This process occurs already during embryogenesis and is crucial for development, differentiation and cellular variability [3], as well as for the transcriptional regulation of genes and miRNA [5]. On the other hand, atypical patterns of DNA methylation are associated with obesity, oxidative stress, hypertension, inflammation, angiogenesis and other pathological processes that are implicated in the development of chronic diseases [6].

Obesity is a systemic disease and a growing health problem that contributes to the increased risk of many common medical conditions [7]. The excess of adipose tissue provokes chronic low-grade inflammation, associated with immunological activation and oxidative stress, insulin resistance, hypertension and dyslipidemia [8]. The most common approach to determine general obesity is body mass index (BMI), the ratio between the mass (weight) and height of an individual, expressed in kg/m^2^. However, clinical evidence suggests that diseases, such as diabetes, are more associated with central obesity, where visceral adipose tissue is stored [9]. Therefore, waist circumference (WC) and waist-to-hip ratio can well account for obesity-related pathologies [10].

Increasing prevalence of obesity worldwide is mostly due to changes in the environment, whereas a person’s genetic profile is considered one of the main causes of individual difference in predisposition to weight gain. A high heritability of this phenotype has been confirmed in several epidemiological studies [11] and examined in genome-wide association studies (GWAS), where 27–30% of the total BMI variance was explained by common single nucleotide polymorphisms (SNPs) [12].

Furthermore, obesogenic environment during *in uterus* development and in early childhood was associated with an increased risk of a range of chronic diseases in adulthood, showing that early environmental influences can cause permanent effects [13]. This interaction of biological and environmental factors is believed to be mediated by epigenetic mechanisms, by which the environmental factors could change gene expression and thus explain the increased prevalence of obesity in the last few decades [12–14]. Epigenome-wide association studies (EWAS) gave the possibility of in-depth insight into epigenetic changes [15]. Several EWAS were performed to study DNA methylation to identify the common variation in the DNA methylome, related to obesity phenotypes, which pointed out several loci, *i.e. HIF3A* [8] *ABCG1* and *CPT1A* [8, 16].

In order to make further contribution to the comprehension of obesity trait, in this study, we are examining the methylation levels associated with central obesity, measured by WC and waist-to-hip ratio adjusted for BMI, in a healthy population. Using healthy individuals can help to avoid the discrepancies caused by extremes of obesity and comorbidity in population-specific cohorts. Our findings provide new insights into genetic regulation of visceral fat accumulation and are presenting a new variant, which may increase the susceptibility to chronic diseases.

## Methods

### Population

210 healthy related individuals from 73 families of the STANISLAS Family Study (SFS) have been enrolled in this study, including 115 adults and 95 children. The SFS is a 10-year longitudinal cohort that includes three visits at 5-year intervals. It involved 1,006 French families from Vandoeuvre-lès-Nancy, France, first that were first recruited between 1993–1995 [17]. All subjects were of European-Caucasian origin, without the presence of chronic disorders (CVD, cancer, diabetes, hypertension etc*.*). Descriptive characteristics are presented in Table 1.

**Table 1:** Population characteristics

	Adults (115)		Children (95)		Males (105)		Females (105)	
	Mean	SD	Mean	SD	Mean	SD	Mean	SD
Age (years)	40.48	7.53	13.15	2.58	27.09	15.14	29.24	14.37
BMI (kg/m^2^)	24.06	3.22	18.43	2.31	21.77	4.17	21.27	3.79
WC (cm)	80.11	10.28	64.15	6.29	76.62	13.08	69.26	8.96
Hip circumference (cm)	97.13	6.27	82.14	10.09	89.38	11.76	91.37	10.3
Waist-to-hip ratio (cm)	0.82	0.08	0.79	0.06	0.86	0.07	0.76	0.05
Obesity (%)	NA		4.2		NA		NA	
Neutrophils	58.07	8.6	49.06	8.57	51.4	9.05	56.59	9.61
Lymphocytes	33.31	9.38	39.79	8.49	37.9	8.61	34.58	10.14
Monocytes	6.65	8.98	6.64	2.84	6.33	2.36	6.97	9.48
Eosinophils	3.43	9.25	3.33	2.58	3.19	2.4	3.58	9.7
Basophils	1.54	9.26	0.59	0.38	0.66	0.4	1.57	9.7

The study protocols were approved by the institutional ethics committees and all subjects gave written informed consent for their participation in the study. For the participants of less than 18 years old, consent was also given for their participation by their parents. Since SFS is a cohort of families, the parents and their children participated in the study and after being informed by the researchers, they all gave written consent for their participation and the participation of their children.

### Data collection

Data and blood samples were collected during the baseline visit of the SFS. All measurements of the clinical indicators were performed in the laboratory of the Centre for Preventive Medicine (CMP) in Vandoeuvre lès Nancy, France. Blood count was performed using standard methodology in the laboratory of the CMP. WC was taken at the midpoint between the lower margin of the last palpable rib and the top of the iliac crest (hip bone). Trained professionals recorded measurements to the nearest 0.1 cm. BMI was calculated by the Quetelet index formula as weight (kg) divided by height^2^ (m)^2^. Data collection has been previously described [17, 18]. Obesity was defined as BMI ≥ 30 kg/m^2^ for adults and for children BMI ≥ 97th percentile for age and sex calculated on French BMI curves [19].

### DNA methylation assay and quality control

Blood samples were taken in the morning between 8 and 9 a.m. following an overnight fast. Whole blood DNA was extracted by the Miller technique [20] and was stored at − 80 °C. Genome-wide DNA methylation profiling was performed using the Infinium HumanMethylation450 BeadChip (Illumina). Methylation ratio (referred to as beta value by Ilumina’s software), is the proportion of methylated by all CpGs (methylated / (methylated + unmethylated) CpGs). Methylation arrays were analyzed and visualized using the R package minfi (version 1.16.1) [21]. Detection *p*-value was generated for every CpG in all samples, indicating the quality of the signal. Poor quality probes were excluded from the analysis using a detection *p*-value cutoff (˃0.05). Probes, missing in more than 5% of samples were excluded from all samples. Background correction and normalization were performed with Illumina background correction and SWAN [22] to all intensity values for a total of 485 512 probes.

We further excluded probes already annotated in HumanMethlyation450 annotation files (probes containing SNPs, sex chromosomes, and a single base extension (SBE) sites). Finally, the probes containing cross-reactive and target polymorphic CpGs were excluded. All downstream analyses were carried out with software R.

### Genotyping and selection of SNPs

Genotyping was performed by the Infinium CoreExome Illumina assay. Significant WC-associated SNPs, located in the same chromosome as the significant methylation sites were selected from the NHGRI-EBI GWAS catalog [23]. These SNPs were extracted from the GWAS data and used in association analyses as candidate genes.

### Statistical analysis

Waist-to-hip ratio and WC were not normally distributed and were therefore transformed to the e-log scale. Individual analyses have been performed for the adult population, the children population and the combined population (children and adults together). A linear mixed-effects model was used to analyze the association between methylation levels at each probe and log-transformed phenotypes. The model used included sex, age, BMI, family structure, and individual white cell counts (neutrophils, lymphocytes, monocytes, eosinophils and basophils) as covariates and chip array as random effect. Bonferroni correction and false discovery rate (FDR) methods (< 0.05) were used for the correction of the results for multiple testing. The association analyses of methylation values and the assessed phenotypes were performed by using the package CpGassoc in R [24]. HumanMethlyation450 annotation file was used for annotating the probes and their corresponding genes. Deviation of multiple correlation squared *ρ*^2^ from constant (random model) of G*Power software was used in order to calculate statistical power [23], specifically the post hoc power analysis procedure. This procedure is parameter analysis which requires the type of test (tails: one or two), effect size (H1 *ρ*^2^ and H0 *ρ*^2^), α error probability, total sample size, and number of predictors.

In order to investigate whether the observed association between WC and methylation level was related to genetic variants associated with WC, a linear mixed-effects model with age, sex, BMI, family structure, methylation batch array and white cell counts as fixed effects, and methylation chip as a random effect was carried out for association analysis using the R statistical package nlme [25].

### In silico analysis

The significant CpGs were localized on the Human genome (GRCh38.p12) using Ensembl browser. In silico gene expression was obtained using the BLUEPRINT Data Analysis portal [26] and GTEx Portal [27].

## Results

**Fig. 1:**
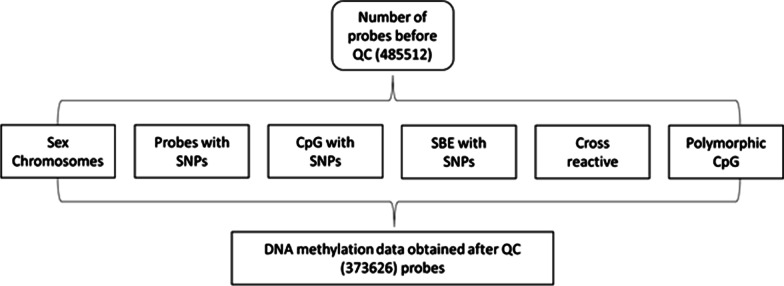
Quality control (QC) processing

Poor quality probes (n = 764) were excluded from the sample. In total, 77% of probes passed quality control, excluding probes containing SNPs, sex chromosomes, SBE sites, cross-reactive and target polymorphic CpGs, thus leaving 373 626 probes for association analyses (Fig. 1). The post hoc analysis of the statistical power of the result was calculated as 100%.

One individual was excluded after quality control checks of the methylation array data (outlier of the plotted median of the methylated against unmethylated samples), thus 210 participants were included in the analyses.

**Fig. 2:**
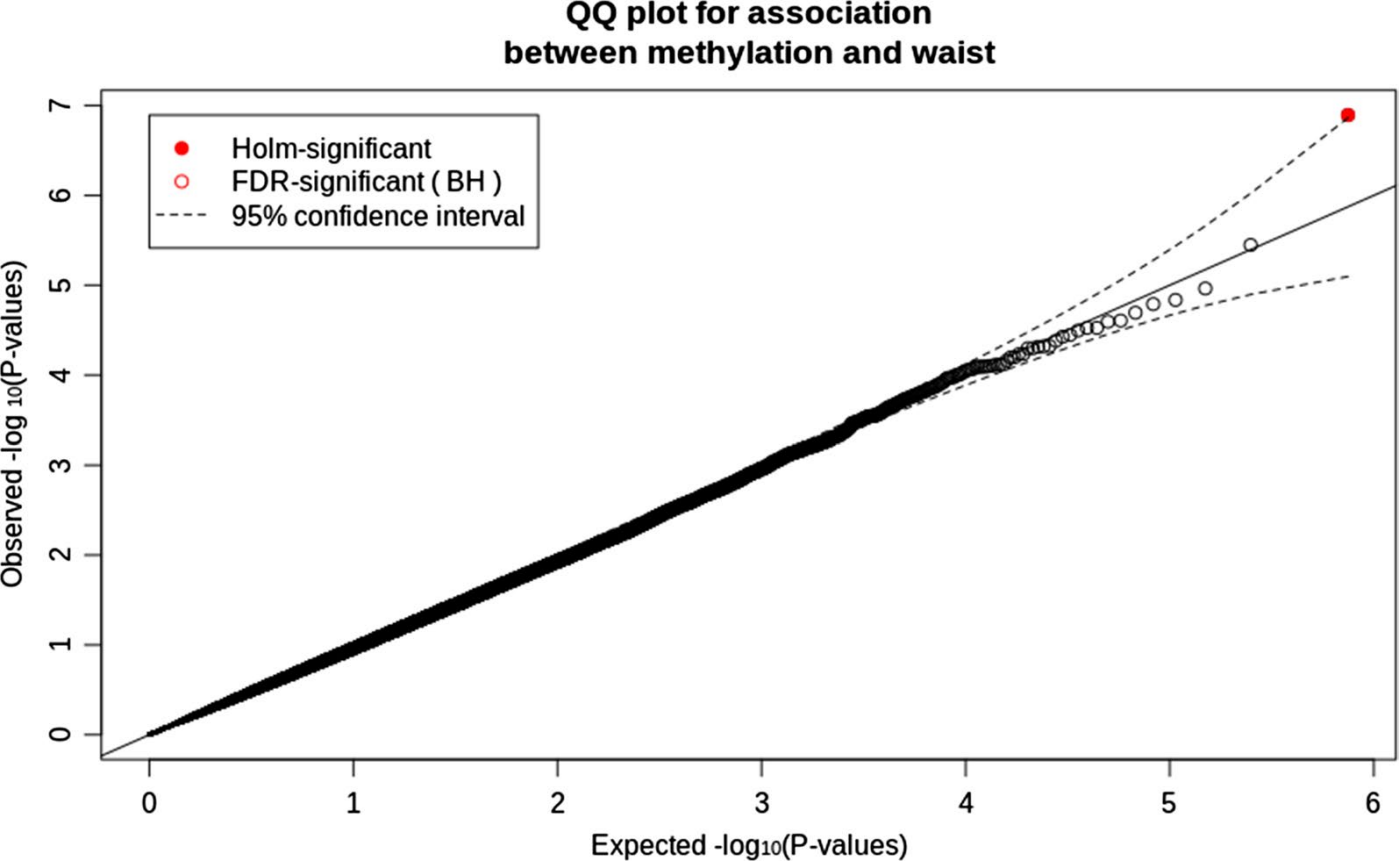
Quantile–quantile plot (QQ plot) of the distribution of observed log10 association *p*-values against the expected null distribution, lambda = 0.937

The results of the analysis showed one novel significant positive association of cg16170243 probe with WC adjusted for sex, age, BMI, family structure, and individual blood cell counts (β = 2.32, SE = 0.41; *P*_*adj*_ = 0.048) in the combined population. QQ plot of the EWAS is presented in Fig. 2. CpG site cg16170243 (chr18:46759502-46759551) corresponds to a 50 bp-length human methylation oligoprobe located on chromosome 18q21.1 (Table 2). No significant associations were identified for waist-to-hip ratio.

**Table 2:** Association of methylation site with waist circumference level, adjusted for BMI

	Children	Adults	All
Effect size	0.01	0.59	2.32
SE	0.009	0.46	0.41
*p*-Value	0.1	0.041	0.13 × 10^−8^
FDR	1	0.95	0.048
Bonferroni	1	1	1

Three SNPs, previously associated with WC in GWAS [28], were identified on chromosome 18 (rs6567160, rs7239883, rs12970134). Minor allele frequencies (MAF) were 0.23, 0.38 and 0.25 accordingly and were in agreement with the MAF in 1000G. No significant associations were identified between these polymorphisms and methylation of the cg16170243.

**Fig. 3:**
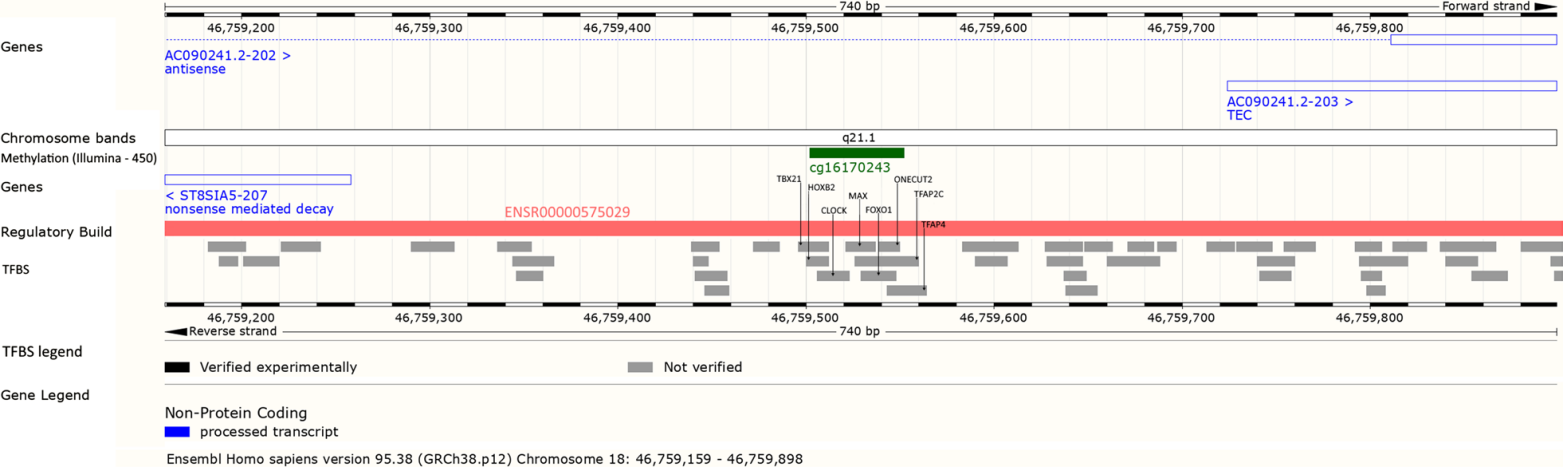
Environment of cg16170243 probe. As depicted by Fig. 3, cg16170243 (in green) is located on chromosome 18q21.1 within intron of AC090241.2-202 transcript and 173 base pairs upstream of the AC090241.2-203 transcript of the forward strand, and 245 base pairs upstream of *ST8SIA5* gene in the opposite strand. Furthermore, cg16170243 is embedded in the promoter flank of *ST8SIA5* gene (ENSR00000575029, in red) and overlaps 8 TFBS (in grey)

The cg16170243 probe maps on chromosome 18q21.1 and is located in the forward strand of the *ST8SIA5* antisense gene (*AC090241.2*), whereas in the opposite strand the probe is located 245 base pairs upstream of the *ST8SIA5* gene (Fig. 3). The *ST8SIA5* antisense gene has 3 splice variants: AC090241.2-201, AC090241.2-202 and AC090241.2-203. Cg16170243 is located both within an intron of AC090241.2-202 and 173 base pairs upstream of AC090241.2-203 (Fig. 3). In silico analysis showed that cg16170243 is embedded in the promoter flank of the *ST8SIA5* gene (ENSR00000575029) and contains transcription factor binding sites (TFBS).

**Fig. 4:**
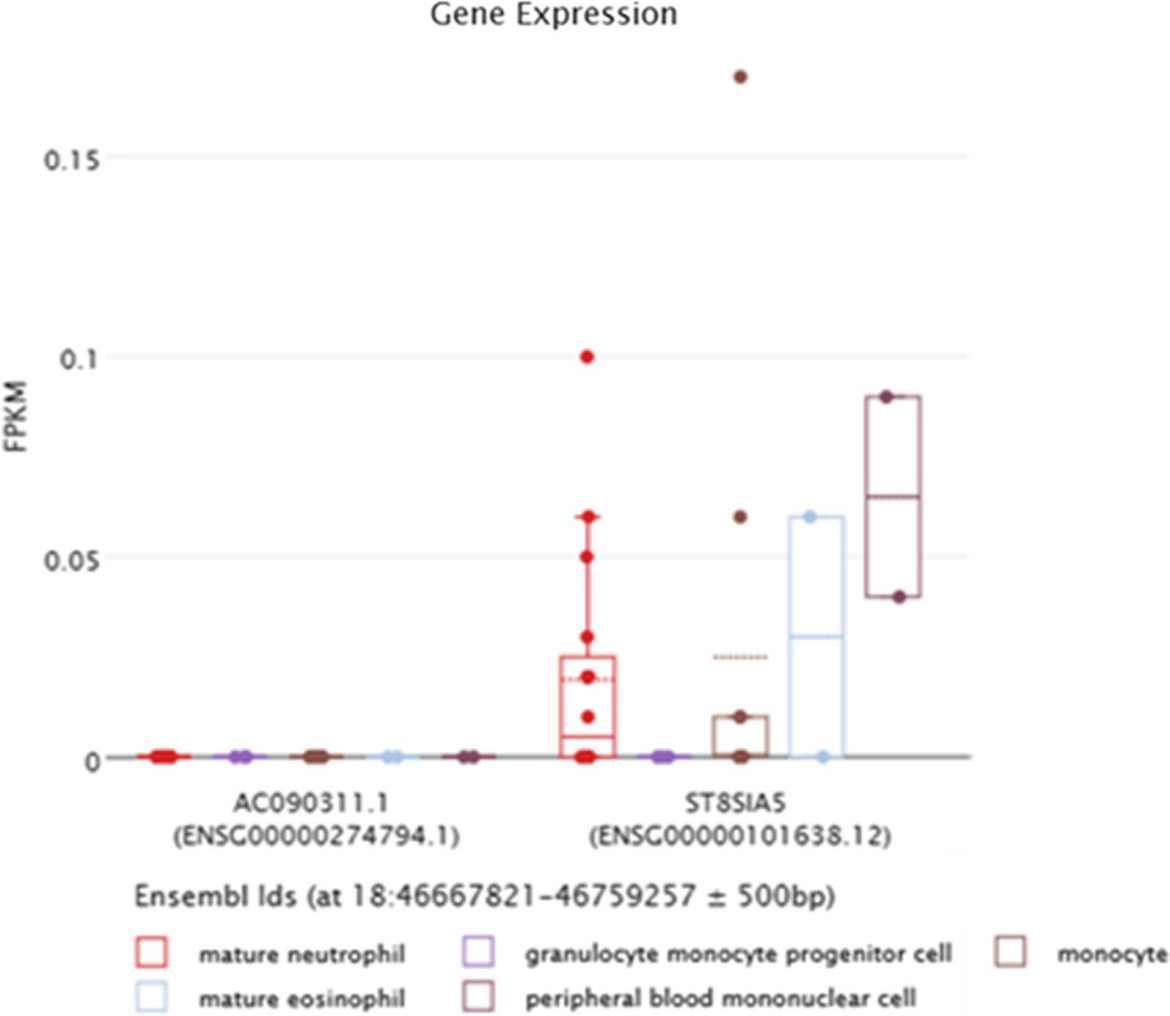
In silico expression of *ST8SIA5* gene and *AC090311.1* pseudogene

**Fig. 5:**
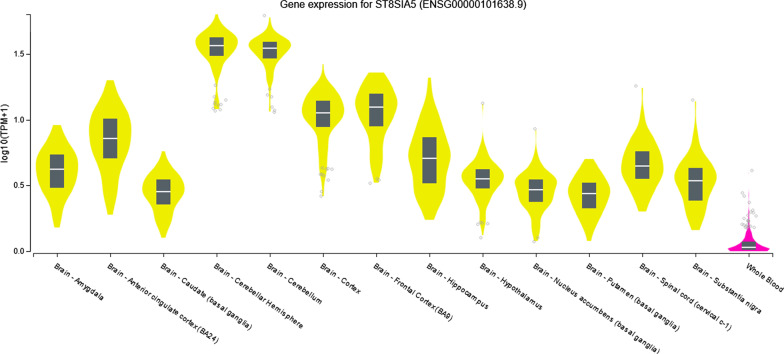
In silico expression of *ST8SIA5* gene in brain and whole blood

Analysis of the expression data showed that *ST8SIA5* gene is expressed in several cell-types of the bloodline: neutrophils, peripheral blood mononuclear cells, eosinophils and monocytes (Fig. 4). Furthermore, the additional data confirmed the expression of *ST8SIA5* in blood cells and indicated that the gene is to a greater extent expressed also in brain structures (Fig. 5).

## Discussion

We have identified a novel significant positive association (β = 2.32, SE = 0.41; P_adj_ = 0.048) between WC, adjusted for BMI, and methylation at the probe cg16170243. There was no association of cg16170243 with three candidate SNPs previously associated with WC. Therefore, the underlying mechanism of the CpG’s effect on WC is probably differentiated from the mechanisms linked to these genetic variants.

The *ST8SIA5* antisense gene has 3 splice variants. Probe cg16170243 is located both in the intronic zone of *AC090241.2-202* variant and at the 5’ of *AC090241.2-203* variant (Fig. 3). *AC090241.2-202* splice variant is an antisense biotype transcript, meaning that it overlaps the genomic span of a protein-coding locus on the opposite strand and can be an important regulator of gene expression [29]. The overlapping gene on the opposite strand of *AC090241.2-202* is *ST8SIA5* (Alpha-2,8-sialyltransferase 8E) gene, coding for a sialyltransferase enzyme, involved in the synthesis of gangliosides GD1c, GT1a and GQ1b from GM1b, GD1a and GT1b, respectively [30]. Gangliosides are glycosphingolipids with one or more sialic acids. They are a component of the bilayer lipid membrane on the cell surface, where they present the points of recognition for extracellular molecules on surfaces of neighboring cells and serve for interaction between cells, adhesion, cell differentiation and transduction of signal [31]. Cg16170243 methylation may modify expression levels of *ST8SIA5* which would lead to an affected synthesis of gangliosides.

Studies showed that an inadequate ganglioside expression in mediobasal hypothalamic neurons deregulates neuronal leptin [32] and insulin signaling [33], which can affect body weight regulation and energy homeostasis. Gangliosides interact with molecules of signal transduction pathways, such as receptors tyrosine kinases (RTKs). Change in ganglioside composition induces the dissociation of RTKs from glycolipid-enriched microdomains, which results in reduced phosphorylation of the receptors and thus causes insulin resistance [34]. Accordingly, insulin is a critical regulator of adipocyte biology and resistance of insulin receptors is, on the one hand, one of the important causes of obesity, and on the other hand, one of the biggest contributors to the development of obesity [35].

Therefore, it is plausible that the effect of cg16170243 on *ST8SIA5* gene in blood cells reflects a process that also occurs on a larger scale in neuronal cells when methylation at cg16170243 site is present, causing a disruption of the insulin signaling pathway and contributing to the accumulation of visceral fat (Fig. 5). Nevertheless, further studies that would confirm such hypothesis are required.

Despite the above-proposed mechanisms of the methylation impact on the WC via insulin deregulation, we cannot exclude the possibility that methylation could be a consequence of modified WC, rather than a cause. Indeed, a meta-analysis from S. Wahl et al*.* has shown that variation in DNA methylation is most often a consequence of adiposity [36]. Thus, particular molecular mechanisms could enable the methylation of regulatory regions of genes involved in obesity, but the exact mechanisms of this regulation remain to be elucidated.

Epigenetics studies of obese phenotypes on healthy individuals have been previously done [37], however, this is in our knowledge the only EWAS study including only healthy subjects. The post hoc power analysis has shown that the statistical power of our result was very high (100%). It should be mentioned, however, that analysis on separate children and adult populations was also performed and no significant associations were identified, probably due to decreased power of the smaller samples of the separate analyses. Similarly, we have performed separate analysis by sex and again no significant associations were identified.

A limitation of our study is the small sample size. Although we have identified a significant result with high statistical power, we did not have the possibility to replicate it in a population-specific study. As epigenetic changes can be tissue-specific, the limitation of our study is also the use of blood samples without tissue-specific replications. Even though the within-subject correlation of CpG specific sites from blood and adipose tissue was previously confirmed, the use of methylation markers in blood to mirror the corresponding profile in the target tissue should be taken with caution [38].

## Conclusions

We identified a novel association between DNA methylation and WC. This association could be due to the modification of the regulatory region of *ST8SIA5* transcription, resulting in a perturbed synthesis of gangliosides.

## Supplementary Information

**Additional file 1**. Peer review reports.

## Data Availability

The datasets generated and/or analysed during the current study are available in the Open Science Framework (OSF) repository at https://mfr.osf.io/render?url=https%3A%2F%2Fosf.io%2F4h5v7%2Fdownload.

## Abbreviations

BMI: Body mass index
CVD: Cardiovascular disease
DNA: Deoxyribonucleic acid
EWAS: Epigenome-wide association studies
FDR: False discovery rate
GWAS: Genome-wide association studies
PBMC: Peripheral blood mononuclear cell
RTKs: Receptors tyrosine kinases
SBE: Single base extension
SD: Standard deviation
SE: Standard error
SFS: STANISLAS Family Study
SNP: Single nucleotide polymorphism
TFBS: Transcription factor binding sites
WC: Waist circumference
