# Supplementary material for: Epigenome-wide association study detects a novel loci associated with central obesity in healthy subjects
Source: BMC Med Genomics. 2021 Sep 23;14:233. doi: 10.1186/s12920-021-01077-9 (PMC8459469; doi:10.1186/s12920-021-01077-9)
Supplement: Supplementary file 1 — Additional file 1. Peer review reports. [file 12920_2021_1077_MOESM1_ESM.docx]

Editor Comments:

I suggest that you revise your manuscript based on the three reviewers' comments.

We operate a transparent peer review process for this journal where reviewer reports are published with the article but the reviewers are not named (unless they opt in to include their name).

Reviewer reports:
Reviewer 1: Please include all comments for the authors in this box rather than uploading your report as an attachment. Please only upload as attachments annotated versions of manuscripts, graphs, supporting materials or other aspects of your report which cannot be included in a text format.
Please overwrite this text when adding your comments to the authors.

This is a study with a important sample size, which assesses the relationship between DNA methylation profile and obesity markers.
Although the topic is relevant, the article is poorly structured. Both the methods and the results are very summarized, little detailed and confusing. It needs to be rewritten and reformulated.

Another important topic is that the waist and hip cincurference ratio is no longer used. Reference 10 is very old (2004) and this concept must be revised throughout the manuscript.

Introduction
1. What do the authors mean by "high nutritional inprit in early life"?
2. According to the authors, what is the difference between genome-wide analysis of DNA and EWAS? Why consider that this is a study of genome-analysis and not EWAS?

Methods
1. The authors considered chronoc disease as CVC or cancer. What about diabetes and hypertension? This information is essential and is not described. It is necessary to include them in the study.
2. The description of the genotyping subtopic is not detailed and clear.
3. In statistical analysis the authors mentioned that "The model used included sex, age, BMI, family structure, and individual blood cell counts". What does family struture mean? Was it evaluated by questionnaire? How all these variables were collected?
4. The description of In silico analyzes already includes a result. How was it really done? All CpG that were significant could go through this analysis? It is not clear.

Results
1. The first result is that children have smaller WC than adults. Wasn't that already expected? Do the authors think it makes sense to make this comparison?
2. It was necessary to show the most characterization results of the patients, in addition to those presented in Table 1. As already mentioned above, assessing the presence of other comorbidities is essential (diabetes and hypertension). Also, were is the results about family structure?
3. Despite showing the average BMI (Table 1), what is the prevalence of obese people in the sample? It is worth remembering that children should be evaluated by the WHO Growth Curves.
4. Don't the authors find it relevant to show the cell count data?
5. The results regarding methylation are not clear. It is not clear whether only a single CpG was associated with WC or whether only it was considered important in the study.
And about the others characteristics of this CpG? is it located in the gene body or in the promoter region? What is the methylation level (beta)?
6. In the methods, the authors refer that the analyzes were adjusted for sex, age, BMI and family struture, but in the results, they report that it was adjusted only by BMI. Considering that the sample includes adults and children and patients of both sexes, the association analyzes also need to be adjusted for these factors, in addition to the BMI. The sample is very heterogeneous and interpretation is erroneous only with adjustment by BMI.
7. The authors show data on gene expression, but this is never mentioned in the objectives or methods. This must be rewritten.

Conclusion
1. Although the authors used expressions like "Eventually" and "could be" in the conclusion, they did not evaluate this cause and effect relationship. Thus, the conclusion must be modified. In my opinion, only the first sentence should be kept "We identified a novel association between DNA methylation and WC. This association could be due to the modification of the regulatory region of ST8SIA5 transcription, resulting in a perturbed synthesis of gangliosides." The other is a discussion and not a conclusion.

Figures
1. The figures are not clear to see (low image quality).
2. Legend figure 6 needs more information.

Abstract
1. The conclusion is not in accordance with the text.


Reviewer 2: Epigenome-wide Association Study detects a Novel Loci Associated with Central Obesity in Healthy Subjects

MGTC-D-20-00034
Xie et al.

This work describes a genome wide methylation analysis for obesity-related traits in nuclear families. The authors find a significant association of one CpG probe with waist circumference (BMI adjusted). The authors report that the probe maps into a gene (AC090241.2) antisense to ST8SIA5 on chr 18. Although this is an interesting finding in itself, the evidence presented in the manuscript and the interpretation of the results do not allow for the conclusions drawn so far. This reviewer summarizes below some major comments that can potentially help to improve the manuscript and the authors may want to take into account.
Major comments:
1. The authors describe that the cohort they used for methylation analyses are 73 families of the longitudinal STANISLAS Family Study (over 10 years with several follow up visits), which is a great resource and was published in other work by the group. It is, however, unclear, from what time point the population characteristics (table1) are and from which corresponding time point the blood samples were taken to perform the DNA methylation analysis. Were blood samples taken at baseline or at follow up visits and to which traits/phenotypes from what visit have these been correlated? This should be explained clearly. Also, as a general comment, there may be a great potential in the clinical follow up data to compare with DNA methylation levels from time point X to explore the longitudinal potential of the population.
2. Table 1. It does not make sense to this reviewer to combine population characteristics between adults and children into a total mean. Same for splitting up in male and female but combined for adults and children…
3. Further, as families have been used, how is this taken into account from a statistical point of view to correct for relatedness? It is mentioned, the model included family structure, however, what kind of approach is not explained in more detail. Further, although the authors describe that the statistical analysis included individual blood cell counts, no data are presented to what cell type generates the detected methylation signal based on their own data set (though an ensemble dataset is presented for ST8SIA5).
4. Figure 2 qq plot: what is the genomic inflation coefficient to quantify the statistical inflation of p-values? No interpretation of this plot is given in the manuscript.
5. It is not clear to this reviewer, how the meta-analysis (table 2, METAL) was performed. It seems, the authors have performed individual methylation analysis for adults and children, respectively and have combined the effect sizes in a meta-analysis. First, this is not described in the statistics part; second, no reason was given for why they splitted up into adults and children.
6. Further with respect to the meta-analysis, to this reviewer it seems a bit odd that the combination of effect sizes (β=0.01 in children with β=0.59 in adults) should result in an overall effect size β=2.32. Also, the p-values presented in table 2 seem similarly implausible. A combined p-value of (not sign in children p=0.1) and (borderline in adults p=0.41) should barely result in strongly significant overall p=0.13x10-8. No effect direction is given. Finally, the corrected p-values for multiple testing (Bonferroni and FDR) are the same, which is unlikely as a 5% FDR p-value should allow for less stringency the Bonferroni correction. The authors may want to take this comment into account.
7. No data have been presented in the manuscript regarding the SNP analyses. Three SNP markers were tested for association with DNA methylation at probe cg16170243. What is the MAFs for the SNPs in that small population and can sign results be expected (from a statistical point of view)? The data should be shown, although these are non-significant. Moreover, the analyses need to be described in statistics section. The authors reference on page10, line 30 to a study reporting the three SNPs and the association with waist circumference; however, Ref 31 is not the original reference but cites the GWAS catalogue.
8. The authors describe, the meth probe falls into AC090241.2, which is located antisense to ST8SIA5. No description of the transcription factor binding sites is given for AC090241.2. Further, it would be worth analyzing corresponding gene expression for both genes to get an impression of functional implications. Further, the authors have published a similar study earlier using the MuTHER cohort to replicate finding. Such analysis in a suitable cohort could strengthen their finding.
9. The argumentation for a potential role of the cg probe and the underlying methylation sites in insulin resistance and central obesity (also shown as a concept model in figure 6) are far too speculative as no data are available and presented to draw this conclusion, especially no data with respect to ST8SIA5 (gene expression, methylation etc.).
10. Another interesting gene located on c18 in the opposite direction (PIAS2, a non-coding RNA functioning as a transcriptional co-regulator) is also a plausible functional candidate gene that might be worth analyzing for gene expression and correlation with clinical traits.
11. Along with these comments the entire discussion is very much focused on ST8SIA5, for which actually no data at all are presented and highly speculative when it comes to drawing conclusion from the data presented. This needs to be "down toned" significantly.

Minor comments:
1. A native speaker should be consulted.
2. The entire abstract needs to be revised. The sentence describing the objective of the study reads "is to use DNA methylation to detect DNA methylation loci …". That should be more specific. Further, the conclusions are actually part of the results. This should be changed. The last sentence is far too speculative and cannot be drawn as a conclusion from the data presented.
3. The introduction is far too long, reading like a textbook. This should be shortened and sharpened.


Reviewer 3: Reviewer comments for BMC Medical Genetics:
manuscript entitled: Epigenome-Wide Association Study Detects a Novel Loci Associated with Central Obesity in Healthy Subjects


The manuscript written by Xie and Gorenjak et al. reported genome-wide methylation data in in human blood samples from the STANISLAS Family Study. The authors performed an EWAS for BMI and WHR but could only detect one CpG site in association with BMI after adjustment for multiple testing.

The manuscript is written in an appropriate manner but needs improvement in some general aspects. Especially the data analysis is very limited and needs some extension.

Major comments:
1. The abstract conclusion is not really a conclusion it's more like a literature review about the identified position. The authors should may focus here on their real study conclusion. The last sentence of the introduction would e.g. represent a possible conclusion.
2. The introduction is too long and partly away from the real important background of the story. E.g BMI explanations aren't really needed. Candidate genes, which never play a role again within the manuscript, do not need a specialfocus in the introduction. Some parts could easily be moved to the discussion section, especially if the group was not able to replicate results from other EWAS this has to be discussed.
3. At which time point was the DNA methylation measured? Would it be possible to analyse association between methylation status and BMI development over time? Since the group has data available over 3 visits?
4. Why did the authors only select SNPs known to be associated to WC and not to BMI and WHR as well? What were the selection criteria for the SNPs?
5. Further, family data over different generations is available in the described cohort. Therefor it would highly improve the study to perform a parent of origin effect analysis on methylation and the influence on the analysed traits as described elsewhere (<https://doi.org/10.1038/s41467-019-09301-y>).
6. Furthermore, the methylation status of the identified CpG could be followed over a generation. This significance of the results would further benefit from this.
7. Although the authors reported that only one association withstand adjustment for multiple testing, the reader would benefit from getting an overview about further results at least in the supplements.
Minor comments:
1. On page 8 line 3: "Biological and clinical measurements for the SFS were collected using appropriate…" The authors describe the measurements with appropriate procedures but didn't even mention what measurement they exactly mean. This sentence is complete meaningless in the way it is now. Please either specify or omit.
2. How would the authors define an atypical pattern of DNA methylation? (page 4; line 37-39).
3. Extending the in silico analysis towards information of chromosomal accessibility at the CpG position would further complete the story.
4. At the beginning of the result section: directly comparing the BMI between children and adults is not the best strategy. It's better to calculate BMI-SDS for children and if needed compare both relative to each other. Further comparing WHR between both is more reasonable than WC.
